# Hepatitis C Virus-Related Central and Peripheral Nervous System Disorders

**DOI:** 10.3390/brainsci11121569

**Published:** 2021-11-27

**Authors:** Rita Moretti, Mauro Giuffrè, Nicola Merli, Paola Caruso, Stefano Di Bella, Claudio Tiribelli, Lory Saveria Crocè

**Affiliations:** 1 Department Medical, Surgical and Health Sciences, University of Trieste, 34127 Trieste, Italy; moretti@units.it (R.M.); paolacaruso83@gmail.com (P.C.); stefano932@gmail.com (S.D.B.); lcroce@units.it (L.S.C.); 2 Department Neurological Sciences, University of Ferrara, 44121 Ferrara, Italy; nicola.merli92@gmail.com; 3 Italian Liver Foundation, AREA SCIENCE PARK, 34100 Trieste, Italy; ctliver@fegato.it

**Keywords:** HCV, hepatitis C, stroke, nervous system, cryoglobulinemia

## Abstract

Hepatitis C Virus (HCV), despite being a hepatotropic virus, is the causative agent of many systemic disorders, such as vasculitis, autoimmune diseases, lymphoproliferative disorders, and a broad spectrum of neurological and psychiatric manifestations. Although symptoms have been misdiagnosed or underdiagnosed, only recently, evidence of direct (inflammatory) or indirect (immune-mediated) HCV-dependent cerebral effects has been established. HCV infection can promote acute inflammatory response, pro-coagulative status and ischemic disorders, and neurodegeneration. These effects rely on cerebral HCV replication, possibly mediated by blood–brain barrier alterations. Further study is needed to better understand the HCV-related mechanisms of brain damage.

## 1. Introduction

The hepatitis C Virus (HCV) virion, first discovered in 1989 [1], is a small (50 nm in diameter) enveloped, single-stranded positive-sense RNA virus that belongs to the family of Flaviviridae and is the sole member of the genus Hepacivirus [2]. HCV infection is a significant cause of chronic liver disease. The worldwide seroprevalence of HCV is estimated to be from 1 to 3%, and according to data from the World Health Organization, in 2015 around 71 million chronically infected individuals worldwide were detected, accounting for 326,000 deaths due to HCV-related liver cirrhosis and 167,000 deaths due to HCV-related hepatocellular carcinoma [3]. HCV seroprevalence varies highly geographically, from 1.3–1.6% in the United States to 15% in Egypt [3].

HCV can be transmitted percutaneously (blood transfusion or needlestick inoculation) and non-percutaneously (sexual contact or perinatal exposure) [4,5]. Following the initial acute infection, HCV may resolve spontaneously. However, around 80% of individuals will develop a chronic infection. In addition to liver damage, HCV infection is related to various systemic disorders, including mixed cryoglobulinemia associated with vasculitis-lymphoproliferative and autoimmune disorders [6,7,8]. Furthermore, chronic HCV infection has been associated with more than 50% of infected patients with neurological and neuropsychiatric disorders [6].

**Figure 1:**
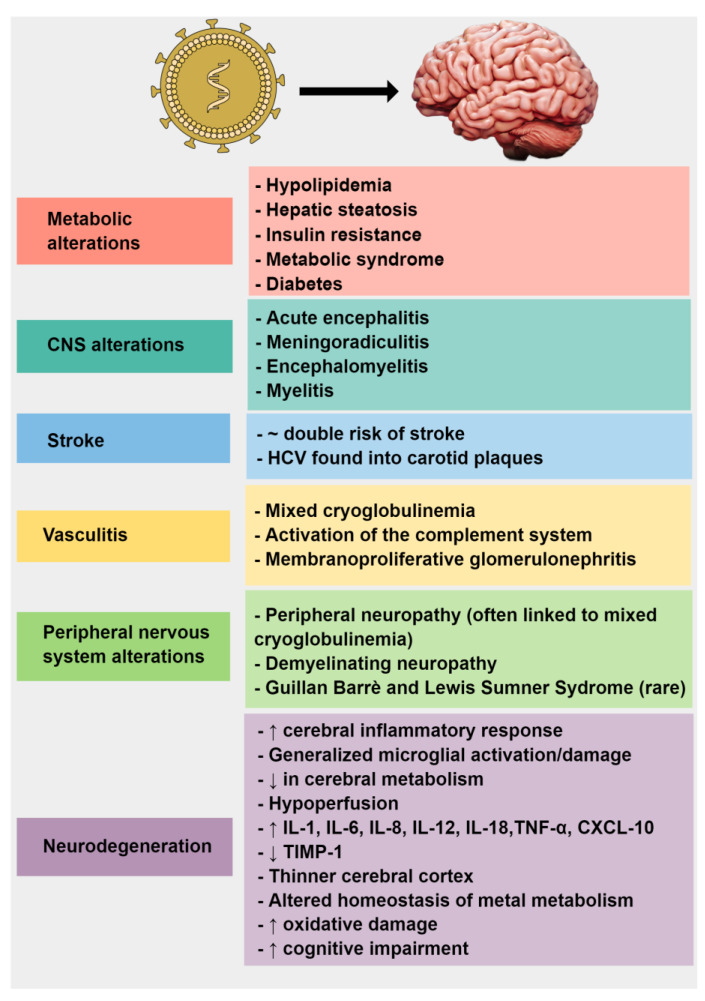
Mind-the-graph: Pivotal correlations between hepatitis C virus and the nervous system.

This narrative review aims to summarize the current evidence that connects HCV infection to the development of neurological and neurodegenerative disorders (Figure 1).

### Search Strategy

A structured search was carried out on MEDLINE/PubMed database employing the following queries alone or in combination with each other: “HCV”, “hepatitis c virus”, “HCV infection”, “chronic HCV”, “central nervous system”, “CNS”, “peripheral nervous system”, “metabolism”, “metabolic”, “cerebrospinal fluid”, “CSF”, “stroke”, “ischemia”, “Mixed Cryoglobulinaemia”, ”cryoglobulineamia”, “vasculitis”, “peripheral neuropathy”, “Parkinson’s Disease”, “Alzheimer’s Disease”, “Amyotrophic Lateral Sclerosis”, “Small Vessel Disease”. Literature retrieval started on 30 June 2021 and was concluded on 15 July 2021 by including articles published up to 15 July 2021, and was performed by two independent researchers with hepatological and neurological experience in HCV infection and with previous experience in bibliographic searches.

## 2. HCV and Metabolic Alterations

Apart from the well-defined hepatic tropism, HCV can induce metabolic alterations promoting hypolipidemia, hepatic steatosis, insulin resistance (IR), metabolic syndrome (MS), and diabetes [9,10,11]. HCV is tightly linked to lipid metabolism and seems to interfere in the lipid and glucose metabolism of the host cells [12,13]. Lipid metabolism could be affected by HCV, due to enhanced lipogenesis, by inhibition of sterol regulatory-element binding protein (SREBP), and by up-regulation of fatty acid synthase (FAS), which is involved in the de novo synthesis of fatty acids [14]. Geranylgeranyl lipid, an intermediate product of the mevalonate pathway of the cholesterol synthesis that is produced during the synthesis of cholesterol, is required for HCV RNA replication [15,16]. Moreover, HCV determines a significant impairment of the mitochondrial lipid B oxidation, with consequent low lipid combustion [9,17]. Furthermore, HCV downregulates the microsomal triglyceride transfer protein (MTTP) activity, causing a reduction in lipid migration from the liver [18]. It has also been demonstrated that HCV core protein localizes in the membrane of lipid vesicles and induces hepatic fat accumulation by activating SREBP-1c [19,20], and interacts with retinoid X receptor-alpha, a transcriptional regulator of lipid metabolism [21]. We should also consider the HCV-induced alteration of lipogenic gene expression through the amino acid substitutions at positions 182 and 186 of genotype 3a (G3a) HCV and amino acid 70/Q of genotype 1b (G1b). That is probably the main reason for the hepatic steatosis induced by genotype 3 HCV [22,23,24].

HCV interacts with glucose metabolism as well. In particular, it downregulates glucose transporter 2 (GLUT2), which transports glucose to hepatocytes; moreover, it upregulates the genes for phosphoenolpyruvate carboxykinase (PEPCK) and glucose 6-phosphatase (G6Pase), which are rate-limiting enzymes for hepatic gluconeogenesis [9,25]. HCV enhances the production of suppressors of cytokine signaling 3 (SOCS-3) [26] and inhibits insulin receptor substrate function to alter glucose metabolism via the downregulation of GLUT4 and the upregulation of PCK2 [9]. In addition, HCV core protein directly influences the proteasomal degradation of insulin resistant S 1 and 2 proteins (IRS1 and 2), thereby interrupting insulin signaling [27]. Therefore, HCV core proteins favor gluconeogenesis and limit glycolysis [13,28,29]. All the metabolic, indirect effects of HCV core proteins, together with the inflammatory process that HCV promotes, should be considered in exploring the impact of HCV on the central and peripheral nervous system.

## 3. HCV and the Central Nervous System

Central nervous system manifestations among patients with HCV infection are uncommon. It is unclear if the CNS can support viral replication. A recent study demonstrated the expression of HCV receptors in brain microvascular endothelial cells, which could allow viral transmigration through the blood–brain barrier [30]. However, viral quantification analyses showed 1000-fold lower HCV RNA concentrations in the brain when compared to the liver and plasma, despite HCV-antigens that were reported in microglia and astrocytes [30]. HCV could lead to various CNS complications ranging from cerebrovascular events to autoimmune syndromes [30].

Inflammatory neurologic disorders comprise encephalic and meningeal inflammation, including acute encephalitis, meningoradiculitis, and encephalomyelitis [30]. Different authors have suggested an association between HCV infection and transverse myelopathy, one of the principal inflammatory conditions studied in HCV patients. It represents an acute or subacute inflammatory disorder, with interesting spinal cord functions resulting in motor, sensory, and autonomic symptoms, depending on the level of spinal cord lesion. The clinical scenario is dominated by sensory ataxia, spastic paraparesis or paraplegia, sensory loss, and urinary and intestinal dysfunctions [31].

Nolte and Colleagues described one of the first cases in patients with active HCV infection, sensory ataxia and HCV RNA detected in cerebrospinal fluid (CSF) specimens [32]. Aktipi et al. reported a case of severe recurrent transverse myelitis in a series of seven patients, all of them with HCV viral activity. However, only two of them manifested with mixed cryoglobulinemia; therefore, it has been hypothesized that an autoimmune pathogenetic mechanism directed against blood vessels or myelin due to chronic viral antigenic stimulation occurs. Additionally, HCV RNA was detected in the CSF of one patient [33].

Suzuki et al. diagnosed a case of acute transverse myelitis clinically manifesting with hypoesthesia below the navel level, paraplegia, urinary retention, and severe constipation, treated with steroid therapy [34]. Moreover, Grewal et al. described a case of recurrent HCV-related myelitis, in which spinal cord biopsy showed demyelination, tissue infiltration by macrophages, and perivascular lymphocytes neither HCV-RNA nor HCV, suggesting antibody-mediated pathogenesis [35]. All of these reports, taking into account the small number of cases, share some standard features that we must consider: the clinical presentation, characterized by initial mild myelopathy with sensory ataxia and later critical motor and autonomic involvement; the detection of HCV RNA in serum and, in some cases, in CSF; mild elevation of proteins in CSF and, occasionally, pleocytosis; brain MRI often normal with concomitant multisegmental alterations of the spinal cord at cervical and thoracic levels; serum cryoglobulins mainly were negative [36].

Moreover, Kitada et al. described a case of a patient with HCV active infection who exhibited longitudinally extensive transverse myelitis with positivity for anti-AQP4 antibodies and concomitant demyelinating peripheral neuropathy in the absence of cryoglobulins [37].

A recent retrospective review by Feldman and colleagues reported that, among 492 patients, 131 had neurological symptoms associated with mixed cryoglobulinemia, while only one patient developed inflammatory involvement of CNS. More specifically, this patient presented a subacute onset of headache, confusion, urinary retention, and paraplegia. In addition, CSF was notable for lymphocytic pleocytosis; MRI showed intracranial and spinal leptomeningeal enhancement with parenchymal signal changes suggestive of meningitis/myelitis and was treated with intravenous immunoglobulins and methylprednisolone, with gradual recovery [38].

Cases of HCV-related leukoencephalitis are documented in rapidly evolving forms with perivascular infiltration of T cells and microglial nodules. For example, Seifert et al. presented a young woman with aphasia, impaired vision, and holocephalic headache; brain magnetic resonance revealed leukoencephalopathy in the parietal and occipital lobe; CSF was found with elevated leukocyte count. Three months later, the patient developed comatose status and tetraparesis. HCV RNA was detected in serum, and brain biopsy showed parenchymal and perivascular T-cell infiltrates, indicating prominent microglial activation; demyelinating aspects were excluded [39].

Acute disseminated encephalomyelitis (ADEM), an immune-mediated inflammatory disorder of the central nervous system that typically follows acute viral or bacterial infections, has also been reported. ADEM is characterized by widespread demyelination that primarily involves the white matter of the brain and spinal cord. Sacconi et al. described the first case of ADEM in a woman (HCV negative) who, 50 days after undergoing a surgical procedure in which she received blood transfusions, suddenly developed seizures, altered consciousness, right hemiparesis hemianopsia, and urinary retention. MRI documented multifocal abnormalities involving gray and white matter of parieto-occipital regions and serum HCV RNA highly positive [40]. A second case was registered by Sim et al., reporting a patient with subacute onset of dysarthria, left-sided hypoesthesia, and right-sided weakness who tested positive for HCV RNA. At the CSF examination, the anti-HCV antibody was positive, and brain MRI showed multifocal signal intensity lesions in the brainstem, thalamus, and cervical and thoracic spinal cord [41]. Both these patients were treated successfully with intravenous corticosteroids.

Lastly, a rare case of the fatal progressive encephalomyelitic syndrome has been illustrated by Bolay et al., reporting a clinical configuration of progressive quadriparesis, paresthesia and numbness, urine, and fecal incontinence associated with muscular rigidity, abnormal postures, and frequent painful muscle spasms. In addition, the post-mortem examination showed encephalomyelitis of the brain, brainstem, and spinal cord affecting both gray and white matter, characterized by perivascular lymphocyte infiltrates, neuronal loss, astrogliosis, and microglial reaction [42].

## 4. HCV and Stroke

HCV infection has been widely reported as an independent risk factor for cerebrovascular-related deaths whose mechanisms remain unexplained. Several case–control and cohort studies reported an increased risk of stroke in patients affected by HCV infection. For example, a large retrospective study that included HCV-positive subjects and HCV-negative control patients, detected an increased risk of strokes in patients with chronic HCV infection (odds ratio (OR) = 1.76; 95% CI: 1.23–2.52) [43]. Data from Liao et al. showed an increased cumulative risk of stroke in patients with HCV compared with people without HCV infection, with an adjusted hazard ratio of 1.27 (95% CI: 1.14–1.41) [44]. Similar data have been published by Hsu et al. [45], who found a higher prevalence of stroke in patients with HCV infection when compared to a control group (26.8% vs. 6.6%), and by Lee et al. [46], who found a hazard ratio of 2.18 (95% CI: 1.50–3.16) for cerebrovascular death in anti-HCV seropositive patients. A recent meta-analysis that included six studies (three conducted in the US, one in Italy, and two in Taiwan) found a pooled OR of 1.97 (95% CI: 1.64–2.30) in terms of risk of stroke and cerebrovascular death in HCV patients vs. controls [47]. In this meta-analysis, special attention has been paid to the mechanisms behind HCV-related stroke pathogenesis. In particular, positive correlations were found between HCV and carotid atherosclerosis/cardiovascular disease, especially in patients with pre-existing conditions, such as diabetes. These correlations can be explained because HCV RNA is present, and inactive replication within carotid plaques occurs [48,49]. HCV core protein has been shown to independently predict the development of carotid plaques [48,49]. HCV-induced local inflammation (in terms of mitochondrial injury and pro-inflammatory cytokine production) may result as a key contributor to plaque instability, leading eventually to its rupture and eventual thrombo-embolism [50,51].

Conversely, HCV eradication has been associated with a lower risk of long-term cerebrovascular events [45,52] and with a reduction in intima-media thickness (from 0.94 mm to 0.81 mm, *p* < 0.001) as compared to infected and untreated patients [53]

Additionally, large vessel occlusion and stroke related to HCV infection may be seen in the context of HCV-induced vasculitides, such as mixed cryoglobulinemia (i.e., precipitation of complement-fixing immune complexes in vessel walls, involving small vessels and cerebral arterioles), ANCA-associated vasculitis, and antiphospholipid syndrome [54,55,56]. On the other hand, it is well-established that HCV infection may increase bleeding risk and intracerebral hemorrhage (ICH). The mechanisms of ICH include pathological changes in the cerebral arteriolar walls. Similarly, persistent inflammation due to viral infection and HCV infection might cause injury in cerebral arterioles, increasing the formation of arterial ectasia and microaneurysms and leading to ICH development. It appears that HCV infection was significantly more frequent in patients with ICH than controls (8.7% vs. 3.5%, *p* < 0.01) [57], and that the risk of ICH was higher in the HCV cohort than in healthy individuals (HR 1.60, 95%CI: 1.24–2.06) in the control group, with an adjusted hazard ratio (aHR) of 1.60 (95% confidence interval [CI]: 1.24–2.06), with an overall increased risk in younger populations if compared to older individuals [58].

## 5. HCV and Cryoglobulinemic Vasculitis

Mixed cryoglobulinemia (MC) is the most documented extrahepatic manifestation. It is characterized by the presence of circulating immunocomplexes produced by the clonal expansion of B lymphocytes. The precise definition is based on laboratory criteria: the presence of abnormal immunoglobulins in the serum that precipitate at temperatures below 37 °C and dissolve by warming the serum. There are three types of cryoglobulinemia, and hepatitis C is most frequently associated with mixed cryoglobulinemia. It is estimated that up to 50% of patients with chronic hepatitis C infection have mixed cryoglobulinemia [59,60,61].

The term “cryoglobulin” was first introduced in the 1940s, when cryoprecipitate proteins were found in patients with multiple myeloma; later, the cryoglobulinemic disease was described in 1966, when Meltzer et al. observed in a group of patients with cryoglobulinemia a joint clinical presentation: purpura, arthralgia, and asthenia, accompanied by organ dysfunction and high concentration of rheumatoid factor (FR). Hence, based on the composition of cryoprecipitate, three serological types of cryoglobulinemia have been identified [59,60,61,62,63,64].

Type I, or simple cryoglobulinemia, consists of monoclonal immunoglobulin serum, generally an IgM or IgG, and usually a paraprotein. It is found predominantly in hematological or lymphoproliferative disorders such as multiple myeloma, Waldenström macroglobulinemia, and chronic lymphocytic leukemia (LLC). Clinically it is often asymptomatic, although serum hyperviscosity syndrome with increased cardiovascular risk, Raynaud phenomenon, and lower limb ulceration are characteristic finds. It represents 10–15% of the cryoglobulinemia forms.

Type II includes cryoglobulins composed of polyclonal IgG with the function of autoantigen and monoclonal IgM. IgM represents the corresponding autoantibody able to exercise rheumatoid factor activity, reacting with the Fc portion of the IgG and determining the formation of an immunocomplex capable of cryoprecipitate. Type II cryoglobulinemia is the most frequent form, comprising 50–60% of the three types.

Type III represents 30–40% of cryoglobulins, and it is characterized by a structure similar to that of type II; the IgG component is, in fact, still polyclonal (as in type II), while IgM is polyclonal as well, always provided with rheumatoid factor activity.

Type II and III cryoglobulins are referred to as MC because of the heterogeneous composition of cryoglobulins, which have an IgG fraction and an IgM component. Moreover, until the early 1990s, mixed cryoglobulinemia was also called “essential” since it was not attributable to a specific etiological agent: it was believed that there was an occasional association with autoimmune, hematological, or infectious pathologies. However, when HCV was discovered in 1989, it quickly became apparent that there was a very close relationship between chronic HCV infection and mixed cryoglobulinemia, and soon multiple studies showed that HCV prevalence in patients with CM, despite geographical variability, stands at 90% and is hugely pronounced in Southern Europe and the Mediterranean areas [65]. Additionally, it has been estimated that circulating levels of cryoglobulin can be found in more than 50% of individuals with chronic HCV infection, although a clear symptomatic presentation manifests in a minority of about 5–20% of subjects [65,66].

Cryoglobulins originate from the clonal expansion of B cells in the context of lymphoproliferative disorders or persistent immune stimulation supported by chronic infections or autoimmune pathologies. Hepatitis C infection is the most studied model for understanding MC pathogenesis: HCV infects lymphocytes and other types of immune cells. Lymphotropism seems to be the critical pathogenic mechanism in triggering multiple extrahepatic manifestations. The etiopathogenesis of mixed cryoglobulinemia is probably a consequence of different and multifactorial steps, including hepatitis C virus (HCV) genotypes and proteins, host factors, and possibly other environmental agents. In addition, HCV may exert a chronic stimulus on the immune system through different core proteins.

Essential is the interaction between HCV envelope protein E2 and CD81, expressed on the surface of both hepatocytes and lymphocytes. Another critical step is represented by the translocation T(14, 18), observed in high frequency in peripheral lymphocytes of HCV-positive subjects with MC; this mutation was later related to the recombination and activation of Bcl-2, a proto-oncogene with anti-apoptotic properties that stimulates and increases the survival of B lymphocytes by inhibiting their apoptosis. The E2-CD81 interaction increases the frequency of gene recombination in B lymphocytes, promoting T(14, 18) translocation and Bcl-2 activation; this proto-oncogene leads to extended B-cell survival. Consequently, B-lymphocyte expansion is responsible for vast autoantibody production. Molecular mimicry between viral epitopes and host autoantigens has also been suggested as part of this multistep process [67,68,69,70]. Ultimately, a recently discovered cytokine belonging to the TNF family, B-cell activating factor (BAFF), would seem to play an essential role in the maturation, proliferation, and clonal expansion of B lymphocytes; the detection of high serum levels of BAFF in patients with LES, AR, SS, and chronic HCV infection strongly suggests the correlation between BAFF and HCV cryoglobulinemia. In support of this evidence, the serum concentration of BAFF was even higher in patients with chronic HCV infection and clinical manifestations of MC [71,72].

The pathogenesis of cryoglobulinemic vasculitis is based on the activity of the complement system, one of the primary regulators of immunocomplexes (ICs). Complement proteins binding to new immunocomplexes control their size and keep them in solution. However, it has been documented that the effective binding of the complement protein C1q with cryoglobulins is a relevant pathogenic mechanism. In particular, HCV core proteins circulate together with immune complexes composed of IgM with rheumatoid factor molecules linked to IgG (exerting an anti-HCV reactivity). HCV core proteins can interact directly with the globular domain of the C1q receptor (gC1q-R), which is expressed on the surface of both circulating blood cells and endothelial cells. This interaction plays an immunomodulatory role by promoting T cells’ proliferative response inhibition, making them unable to suppress self-reactive B cell clones with FR activity. Moreover, this mechanism could facilitate the deposition of immunocomplexes and induce vascular inflammation, leading in some cases to leukocytoclastic vasculitis. It represents the histopathological hallmark of cryoglobulinemic vasculitis [73,74,75].

The clinical syndrome of MC is characterized by a wide variety of symptoms and is generally considered a systemic vasculitis affecting small blood vessels or, less frequently, medium-caliber vessels. The suggested etiopathogenesis of the damage appears to be related to the recruitment of leukocytes in the vessels and the deposition of immune complexes on the vascular walls, with subsequent inflammation, activation of the complement system, and promotion of microvascular injury [60,76].

Skin involvement is one of the most common clinical manifestations of cryoglobulinemic vasculitis: palpable purpura is the key symptom, present in most symptomatic patients (54–82%) as the first sign of the disease. This presentation can be accompanied by arthralgia, asthenia, fever, or myalgia; such symptoms, when combined with purpura, strongly suggest vasculitis. Raynaud’s phenomenon and livedo reticularis are also frequent. Petechial lesions are usually of small size and are generally found in the lower limbs, but can also extend to the abdominal region and upper limbs; in the most severe cases (10%), skin ulcerations can be observed at the malleolar level, resulting in a risk of necrosis and infection. The fundamental role of HCV in vasculitis pathogenesis is supported by the detection of viral protein deposits in skin blood vessels, vascular walls, and perivascular spaces of cryoglobulinemic patients. IgM and IgG, along with complement proteins, have also been demonstrated at vascular damage sites [77].

Joint manifestations (44–71% of cases) consist predominantly of bilateral and symmetrical pain localized to the hands, knees, and wrists, without signs of inflammation, swelling, or deformation of the joints. To these symptoms are added asthenia and fatigue in more than 60% of patients [78].

Finally, renal involvement occurs in 30–60% of patients with MC, often connected to cutaneous vasculitis. It is mainly characterized by membranoproliferative glomerulonephritis, endocapillary proliferation, macrophage infiltration, and subendothelial deposits of IgM, IgG, and C3. Symptomatology typically consists of different proteinuria levels, microhematuria, hypertension, and moderate renal failure, while acute nephritis and nephrotic syndromes are observable in less than 20% of cases [79,80].

## 6. HCV and Peripheral Nervous System

The most documented and recurrent manifestation in patients with chronic HCV infection is peripheral neuropathy (PN). The great variety of neuropathies is primarily related to MC, with a prevalence of 36–86%. Sometimes PN can be the first clinical sign of the disease [81].

Electrophysiological studies and nerve biopsies have revealed that peripheral neuropathy is associated in most cases with an axonal degeneration process related to cryoglobulins. Nerve injury is believed to be secondary to two main pathogenetic mechanisms: on the one hand, the alteration of microcirculation of the vasa nervorum due to the intravascular deposit of cryoglobulins and consequent vessel obstruction and ischemia; on the other hand, necrotizing vasculitis induced by longstanding precipitation of immune complexes composed of cryoglobulins, activated complement, FR and viral proteins. These are the two mechanisms currently proposed for the etiopathogenesis of axonal damage. In addition, neuropathological data disclose perivascular and interstitial inflammatory infiltration of mononucleate cells in perineurial space, and hyperplasia of the muscle and endothelial layers is found at the vascular wall level [82,83].

The role of the hepatitis C virus is suggested by pathological and molecular studies of nerve biopsies. It has been demonstrated by a non-replicative positive strand of HCV RNA in epineural cells of patients with peripheral neuropathies. Furthermore, it has been associated with inflammatory lymphocytic and monocytic infiltrates and vasculitis of epineural arterioles; however, no genomic negative HCV RNA has been detected so far, demonstrating the lack of viral replication in the peripheral nervous system [84]. Therefore, axonal degeneration is the preeminent lesion in vasculitic neuropathy.

Clinically, peripheral neuropathy’s most regular expression is sensory or sensorimotor symmetrical or asymmetrical distal polyneuropathy, involving principally the lower limbs with acute, chronic, or, more frequently, subacute onset. Alternative clinical presentations include mononeuritis or multiple mononeuropathies, even though in a minority of cases and with a more rapid onset [85].

Patients usually present sensory symptoms, typically sensory loss, paresthesias, dysesthesias, impaired temperature, neuropathic pain, and burning feet with night exacerbation. These manifestations can often precede motor deficits, muscle weakness, and cramps by a few months or years. The impairment of sensitive fibers occurs primarily at the level of small nerve fibers Ad myelinated and C demyelinated; conduction studies and electromyography demonstrated short fiber sensory polyneuropathy (SFSN), a tremendously painful condition characterized by burning in feet, tingling, and restless leg syndrome. Large fiber sensory polyneuropathy (LFSN) seems less frequent, involving larger nerve fibers responsible for proprioception and leading to sensitivity disorders and muscle cramps, numbness, or sensory ataxia [86].

Gemignani et al., analyzing a cohort of 71 patients with cryoglobulinemic neuropathy, found a correlation between the grade of activity of cryoglobulinemia and the severity of neuropathy. SFSN was more common in patients with mild cryoglobulinemic vasculitis, while mononeuritis multiplex or sensorimotor neuropathies were linked to the active cryoglobulinemic syndrome in patients with intense purpura and cryocrit > 5% [65]. This evidence, however, appears not in keeping with data shown by Biasiotta et al., who observed an association between the development of peripheral neuropathy and the duration of HCV infection [87]. Cranial involvement is rarely demonstrated, although Nemni et al., in their case series, also reported a subgroup of patients with cranial neuropathies [88].

Peripheral neuropathy has also been described in patients with chronic HCV infection but without mixed cryoglobulinemia, even with a lower prevalence and less severity. In this context, the most common neurological manifestations are mononeuropathy and multiple mononeuropathies, with both sensory and motor involvement [88]. Additionally, Lidove et al. confirmed these findings in their case series, revealing a prevalence of mononeuropathy multiplex and axonal neurodegeneration associated with perivascular lymphoid infiltrate at neuromuscular biopsy [89].

Sampaolo et al., studying nerve biopsy specimens from 19 patients with and without MC, observed a clear predominance of axonal degeneration pattern in subjects with HCV-related MC neuropathy, whereas myelin alterations were detected in MC-negative patients. The etiopathogenesis in patients without MC is probably characterized by a direct nerve cytopathic effect of HCV or an immune-mediated mechanism based on a possible interaction between HCV and anti-HCV antibodies. The link between HCV and demyelinating neuropathies could be connected to a virus-triggered autoimmune mechanism; it has been hypothesized that the deposition of immune complexes on the myelin surface or cross-reaction of autoantibodies against essential myelin proteins could lead to progressive destruction of the myelin function. The absence of cryoglobulins does not preclude immune complex formation since it is believed that HCV envelope proteins are responsible for complement activation and immunocomplex deposition [90,91].

Demyelinating neuropathies are rarely described in HCV infection and considerably less frequently in patients with mixed cryoglobulinemia, as indicated in the cohorts studied by Moretti et al. and Nemni et al., and are always associated with concomitant axonal degeneration [92].

Authier and colleagues, examining 30 patients with HCV-associated peripheral neuropathy, identified only two cases of chronic inflammatory demyelinating neuropathy (CIDP), an absence of circulating cryoglobulins [93].

Other remarkable cases reports described two female patients with type II MC HCV-related with sensory and sensorimotor neuropathies, presenting on electrophysiological investigations both axonal degeneration and moderate demyelinating pattern, but not satisfying the criteria for CIDP [93,94].

Evidence of CIDP also has been documented by Chin et al. and Tsuzaki et al., who reported cases of patients diagnosed with CIDP and treated with intravenous immunoglobulins, most of whom had significant improvement [95].

Demyelinating neuropathy with monoclonal IgM anti-myelin-associated glycoprotein (MAG) antibodies was demonstrated by a case series of 59 patients investigated by Mariotto et al., who identified three subjects, tested harmful for CGs and RF, with anti-MAG activity [96].

CIDP has also been described as an uncommon side effect in patients treated with IFN-α. Furthermore, other studies illustrate the worsening exacerbation of peripheral neuropathy during interferon therapy. The reason for these events is not yet known, but they are presumed to be due to immune-mediated phenomena [97].

Guillain Barré Syndrome (GBS), an acute demyelinating polyneuropathy, has seldom been described. As far as we know, only seven cases have been reported, with the first ones in 1993 by De Klippel et al. and in 1998 by Lacaille and Colleagues [98,99]. More recently, in 2019, Chlilek et al. reported a case of a male patient presenting quadriplegia, sensory disorders in legs, and areflexia; an electromyographic study demonstrated severe acute motor-sensory axonal neuropathy (AMSAN) with demyelinating lesions, compatible with GBS [100].

Finally, a case of Lewis Sumner Syndrome, a rare and multifocal demyelinating neuropathy, was documented by Caporale and colleagues in 2005, demonstrating a sensorimotor demyelinating neuropathy with persistent conduction block, with asymmetric clinical involvement of sensory and motor nerves [101].

## 7. HCV and Neurodegeneration

Inflammation and necroptosis are two of the most relevant events in neuropsychiatric disorders such as Parkinson’s disease (PD) and Alzheimer’s disease (AD), as well as in amyotrophic lateral sclerosis (ALS) or small vessel disease-related dementia (SVD) [102,103,104,105,106]. Twenty years ago, a famous epoch began when the immune response in brain studies was described as an ongoing and still-actualization, the “inflammaging” [107], one of the most important etiological factors in age-related neurodegeneration [108,109], characterized by a chronic status of low-grade and nonspecific inflammatory status as standard in older brains, with a constant elevation of Il-6, TNF, and IL-1β, and related to a significant decrease in neurons and the rarefaction of the neural network progression, reducing spine sprouting [108,109,110,111,112,113,114,115].

Viruses can injure neurons by direct killing, cell lysis, and by inducing apoptosis [116,117]. Regardless of the immune-privileged status of the CNS, it is still well known that dynamic immune and inflammatory responses result from a variety of insults in this compartment, or a summation effect of them, all along with lifespan, and viral infection accounts for one of them [118,119,120]. Generally, the acute neuroinflammatory response includes an immediate and short-lived activation of the innate immune system within the CNS [121]. This type of response is favorable to the CNS; usually, it is rapidly activated and offers a practical, even if not always without side effects, reparation [120]. On the other hand, whenever there is a chronic condition of inflammation, due to the altered immune response, microglia are activated continuously, with many different consequences, such as the constant release of pro-inflammatory mediators, oxidative damage, and endothelium impaired activation, and frequently all these conditions perpetuate and expand the inflammation process [122,123,124].

HCV is associated with a chronic inflammatory condition in the CNS. Even though HCV primarily targets hepatocytes, it has been discovered in peripheral blood mononuclear cells, cerebrospinal fluid, and in the brain of chronically infected patients [125]. Most reports supporting HCV in the CNS have used PCR-based approaches to detect viral genomes in brain tissue and cerebral spinal fluid (CSF) [105]. Furthermore, it has been ignored whether the virus can be transferred from the periphery to CNS [126]. It has recently been hypothesized that viral replication inside the brain could be possible, as negative-strand HCV RNA, a replicative intermediate, has been found in the CNS, suggesting viral replication. However, the question is still under debate due to the small numbers of patients included in the studies and the objective difficulties in detecting HCV in brain tissue.

Nevertheless, many studies are ongoing, and recently, HCV RNA has been quantified in multiple samples by contemporary studies of the brain and liver of infected patients [127]. Unfortunately, at the moment, there has been no direct and robust determination of viral replication inside the brain, even if Radkowsky et al. [128] have demonstrated in a single case that HCV NS3 sequences isolated from a variety of brain regions were similar to the ones obtained from lymph nodes but very different from serum-derived virus, suggestive of a possible independent viral evolution inside the brain [128]. Furthermore, variability in the HCV internal ribosomal entry site, the so-called IRES, compared to liver sequences, was found in the brain tissues of two HCV patients [129]. Moreover, in the 13 brains of HCV patients, it was found that there was a brain-specific mutation employing a single nucleotide polymorphism that constituted 10% of the HCV sequences in the cerebellum and the medulla [129]. This finding adds another support to the hypothesis of possible HCV replication inside the brain.

The association between HCV infection and significant cerebral inflammatory response is demonstrated by the marked increase in brain metabolites such as choline, creatine, and inositol [130]. Higher choline levels have also been demonstrated in the brains of HCV-infected patients affected by chronic severe fatigue [131]. The spectra of N-acetyl aspartate (NNA) and NNA-glutamate, related to neuronal density and nitrogen removal, were also significantly higher in the basal ganglia of HCV patients with brain involvement [132]. Recent and sophisticated studies employing proton magnetic resonance spectroscopy and positron emission tomography showed a generalized microglial activation related to HCV viremia and a decrease in cerebral metabolism with altered basal ganglia myoinositol/creatinine and choline/creatine ratios [133], as metabolic markers of inflammation [134]. A different study, which recruited 22 HCV patients, 15 treated with pegylated interferon and ribavirin, and seven untreated, demonstrated that treated patients who responded to therapy showed decreased cerebral levels of choline and myoinositol [135]. HCV-related brain dysfunction is accompanied by neuroimaging evidence of white matter neuronal loss, alterations of several commissural and association tracts, cortical hypoperfusion, and basal ganglia hypoperfusion [136]. Moreover, HCV-related patients with associated signs of cirrhosis showed more robust signs of widespread alterations of the white matter, and with focal cortical damage, and they could be based on a chronic neuro-inflammatory response and hyperammonemia effects [137,138].

A post-mortem study of the brains of HCV patients demonstrated significantly increased levels of IL-1, TNF-a, IL-12, and IL-18 [139], which could explain glial cell activation reported in other HCV patients with severe neuropathology, which is not dominant in all the brains examined. By implementing technologies using laser capture microdissection, it has been demonstrated that HCV RNA was present in the microglia and the astrocytes [140]; on the contrary, immunohistochemical studies failed to show specific viral carriers and receptors [141,142]. This suggests that the virus can be a guest of microglia and astrocytes without entering the cell, contrary to what is observed in vitro where neuroepithelioma cell lines express all the molecules required for virus entry: scavenger receptor B-I (SR-BI); tetraspanin CD81 and tight junction proteins claudin-1 and occludin [143]. This report is in clear contrast with another report, which showed limited evidence for HCV entry or replication in various immune cell types [144]. The differences could be based on a less differentiated neural evolution pattern than the standard and efficient neural population [145]. Another study identified the brain cells harboring HCV virions as CD68-positive cells (macrophages/microglia) using two different approaches (anti-NS3 monoclonal antibodies and laser capture microscopy of autopsied brain tissue) [146].

Another convergent opinion is based on the brain microvascular endothelial cells (BMEC), the blood–brain barrier’s major component. They support HCV infection in vitro [126]. BMEC was permissive for cell culture-derived HCV (HCVcc), showing a spreading infection. Viral infection persisted in Huh-7 cells but did not last in BMEC for more than 120 h. The infection, which passed through BMEC, gave significant cytopathic effects and an acute lytic infection together with BMEC cell apoptosis and a reduction of brain–barrier activity after the end of the infectious time, that could also occur in vivo [126].Moreover, a recent study has shown the presence of HCV receptors (including CD68, CD81, claudin-1, LDLR, and scavenger receptor-B1) on the brain microvasculature, representing a gate through which the virus can infect brain cells [126]. A controversial point has to do with mitochondrial dysfunctions, which many different works suggest, proposing that mitochondrial impairment can lead to metabolic modifications, altered response to oxidative stress, calcium signaling, and apoptosis. These data are pretty well-demonstrated in vivo in hepatocytes, but they have never been shown, if strongly argued, in the brain tissue [147]. Multiple lines of evidence delineate the neurobiology of HCV; it is currently unclear how commonly HCV can be found in the brain, what factors promote brain invasion, and the clinical sequelae of this brain invasion [148]. It is still argued whether patients with clinical evidence of HCV have CNS dysfunction either caused or worsened by the virus’s presence [149,150,151]. HCV can induce microglial damage to brain cells through immune cross-reactivity between HCV peptides and brain tissue antigens [152]. Infected/stimulated macrophages and microglial cells release neurotoxins, such as nitric oxide and pro-inflammatory cytokines including tumor necrosis factor (TNF)-α, interleukin (IL)-1, and IL-6 [153]. A midbrain culture from the brain of HCV-positive Wistar embryonic rats showed elevated levels of pro-inflammatory chemokines, such as intracellular adhesion molecule-1 (ICAM-1), regulated on activation normal-T cell expressed and secreted (RANTES), and LPS induced CXC chemokine (LIX); at the same time, there was a reduction of neuroprotective chemokines, such as tissue inhibitor of matrix metalloproteinase-1 (TIMP-1) [154]. ICAM-1 produced by cerebral astrocytes stimulates the TNF-α inflammatory pathway, causing dopaminergic neuronal damage [155].

Similarly, the increased production of LIX and RANTES by primary astrocytes and microglia can induce neuronal apoptosis and demyelination [156,157]. On the other hand, HCV was shown to suppress the expression of TIMP-1, which inhibits matrix metalloproteinase (MMP) [158,159]. The presumed mechanism of brain HCV infection is thought to resemble that found in human immunodeficiency virus (HIV) infection [160,161], where an indirect parameter of viral-caused neurodegeneration can be demonstrated in vivo by a decrease in cerebral cortical thickness [162]. Direct exposure of human neurons to HCV core protein is neurotoxic, with conspicuous signs of microglial activation, neurite retraction, and a general pro-inflammatory status, leading to a dementia-like pattern [140]. Therefore, an interesting study [160] explored cortical thickness in HCV patients, measured using a surface-based approach. Different volumes of whole gray matter, white matter, and cerebellum in HCV patients and healthy control were not found. On the contrary, HCV patients displayed significantly thinner cerebral cortex in multiple areas in both cerebral hemispheres, and, after correction for multiple areas of comparison, in the left frontal lobe (the anterior aspect of the central sulcus) and left and right occipital lobe when compared to the healthy controls [160].

Many studies have also been focused on the simultaneous co-infection between HCV and human immunodeficiency virus type 1 (HIV-1) and HCV co-infection [163]. HCV has been identified in various nervous system tissues and fluids, both in mono-infected patients and those who are co-infected with HIV [150]. HCV co-infection is associated with accelerated HIV disease progression, worsened clinical outcomes, and increased mortality [164]. HIV/HCV co-infected patients have higher HCV levels and a lower likelihood of spontaneous HCV clearance, together with faster progression to liver cirrhosis [163]. It seems that HIV/HCV co-infected patients are more prone to have severe neurological consequences [164,165]. The most relevant and recent work on the topic is the one by Pfefferbaum et al. [166], a longitudinal study conducted over 14 years in patients and control subjects, conducted to examine the effect of HIV and HIV/HCV co-infection on premature aging, non-accelerated differences, or accelerated regional brain volume declines above aging trajectories, compared to controls. As evidence, HIV subjects showed a more consistent decline in the frontal and posterior parietal cortex despite anti-retroviral therapies [167], without significant sex differences [168], but strongly able to interfere with daily living habits [169]. In this study, the most important finding was the real effect of HCV co-infection [170]. Effects of HCV co-infection on lobar volume region relations will emerge, particularly considering the insular, cingulate, parietal, and temporal cortices relative to their HCV-seronegative counterparts and control subjects [169]. Experimental studies [169] have already been conducted, with the infection of human microglia, astrocyte, and neuron cultures with cell culture-derived HCV or exposed to HCV core protein with or without HIV-1 infection or HIV-1 viral protein R (Vpr) exposure. These studies showed that HCV-encoded RNA and HCV core and non-structural 3 (NS3) proteins were detectable in human microglia and astrocytes infected with HCV. HCV core protein exposure induced pro-inflammatory cytokines, including interleukin-1b, interleukin-6, and tumor necrosis factor-a in microglia, but not in astrocytes [169]. On the contrary, increased levels of interleukin-8 and CXCL-10 have been detected in both microglia and astrocytes [169]. HCV core protein modulated neuronal membrane currents and reduced both b-III-tubulin and lipidated LC3-II expression [169]. Neurons exposed to supernatants from HCV core-activated microglia exhibited reduced b-III-tubulin [169]. HCV core protein neurotoxicity and interleukin-6 induction were potentiated by HIV-1 Vpr protein, and HIV-1 Vpr transgenic mice implanted with HCV core protein showed gliosis and reduced neuronal counts together with diminished LC3 immunoreactivity [169]. HCV core-implanted animals displayed neurobehavioral deficits at days 7 and 14 post-implantation [169]. Therefore, animal and cellular studies demonstrated [169] the clinical concerns: HCV core protein exposure caused neuronal injury through suppression of neuronal autophagy in addition to neuroimmune activation, and there was a clear additive neurotoxic effect of HCV and HIV [169,170,171]. The Manhattan HIV Brain Bank (MHBB) and Manhattan Hepatology Brain Bank are well-suited to examining these phenomena. Targeted to patients with either advanced-stage HIV or liver disease, there is a high HCV infection rate in these patients [172]. Therefore, 20 patients who were accepted to be studied post-mortem were recruited: 10 HIV-infected patients had serologic evidence of past or current HCV infection (presence of antibody to HCV) before demise. Nine of these patients had chronic HCV infection as indicated by plasma HCV load. Ten HIV-infected patients did not have detectable HCV antibodies. One of these 10 HIV-positive, anti-HCV antibody-seronegative patients had chronic HCV infection, as determined by HCV plasma load. Of the 10 HIV-naïve patients, five were seropositive for anti-HCV antibodies, and 3 of these five had HCV RNA [71]. None of the HIV/HCV-coinfected patients had received therapy targeted to their HCV infection. Brain HCV was detected in 6 of the 10 HIV/HCV-coinfected patients and 1 of the 3 HCV mono-infected patients. All HCV sequences obtained in this study were genotype 1 except for those from the liver and plasma of one patient, genotype 3a, and one other with genotype 1 b [148]. Six of 7 patients with brain HCV, 6 of 6 patients with liver HCV only, and 10 of 17 patients with no autopsy evidence of HCV sequences displayed severe hepatic fibrosis or cirrhosis. It should be said that there were multiple superimposed risk factors (such as substance/alcohol abuse) for liver disease [148]. Considering all of these variables, the most frequent neuropathological finding was Alzheimer type 2 gliosis (6 of 12 patients with HCV and liver fibrosis/cirrhosis) compared to those with HCV-negative fibrosis/cirrhosis (3 of 10 patients), an apparent enlargement of astrocyte nuclei, likely related to metabolic alterations that were liver-related [148]. There was no evidence of HCV- or HIV-associated encephalitis. PCR for HIV DNA was negative in the brains of all patients with HCV [148].

In 2015, data from the Keelung community-based integrated screening (KCIS) program regarding the prevalence of HCV infection, PD, and other confounding variables among the Taiwanese population were developed [154]. They showed a statistically significant association (adjusted odds ratio = 1.39, 95% CI [1.07–1.80]) between PD and HCV infection. They assessed HCV dopaminergic neuronal toxicity at the molecular level compared with that of 1-methyl-4-phenylpyridinium (MPP+), typically employed in experimental conditions, to develop PD, and they acted in the same way. Some other data [171] stressed the relationship between HCV, PD, and younger patients (less than 65 years old). Still, it cannot be denied that there is a greater risk of drug use in this population, which might be considered a summary risk factor for PD development. However, these results are not in line with other studies [172], mainly due to geographical and ethnographic reasons [171].

Another important marker of neurodegeneration is the altered homeostasis of metal metabolism, such as that of copper, iron, and manganese [173]. They usually participate in different biological processes, but when there is altered catabolism, their accumulation leads to oxidative damage [51], which frequently occurs in other brain structures, i.e., the basal ganglia, mostly affected due to their high metabolic rate [174]. The metal metabolism relies on the liver production of different proteins, i.e., transferrin (Tf) and ceruloplasmin (Cp). Ceruloplasmin has an intrinsic ferroxidase activity (ECP) [174], and it helps oxidize the ferrous iron to ferric iron, which can be charged on Tf, then delivered to various organs [175]; moreover, Cp regulates the manganese transport system. Finally, Tf and Cp’s combined actions cooperate to act as metal scavengers, particularly ferrous ions, leading to potentially fatal oxidative reactions. Apart from the genetic alterations of ceruloplasmin (such as in aceruplasminemia or Wilson disease), there are many observations of altered brain accumulation of metals in many neurodegenerative disorders [176].

Moreover, hepatic encephalopathy is associated with a constant increase in ammonia, together with astrocyte swelling and microglia activation [177]. Nevertheless, there is also a constant, elevated deposition of manganese and copper in the basal ganglia, with in vivo demonstration by MRI studies, which identified T1 hyperintensities in globus pallidus. Astrocytes, which are the first victims of hepatic encephalopathy, produce Cp in a membrane-anchored isoform that lets cellular iron efflux; nothing is known about what happens during the chronic disease progression damage induced by hepatic encephalopathy [178]. Recent studies have demonstrated that chronic hepatic encephalopathy produces a constant decrease in globus pallidus and caudate volumes, directly correlated with its volume reduction and ceruloplasmin with ferroxidase activity (also called ECP) levels and inversely correlated with the pallidal volume [174]. The altered ECP leads to reduced iron oxidation and chelation by Tf, incrementing ferrous ion levels and altered oxidative stress-induced damage [174,175,176,177,178].

The clinical evidence of what has been reported above is that cognitive impairment, or difficulty with thinking, has been well-documented in persons with chronic HCV and, until recently, was assumed to be a consequence of cirrhosis-associated hepatic encephalopathy (HE). Conditions such as portal–systemic shunting can result in cerebral dysfunction, hallmarked by decreased recent memory, fluctuating consciousness, and disorientation thought to be an outcome of high ammonia concentration and astrocyte swelling [179]. Many of the cognitive syndromes might be derived from a diffuse involvement of the white matter of HCV-infected patients, determined by cryoglobulins’ co-existence and circulating anti-cardiolipin antibodies. The diffuse white matter alterations are particularly evident in periventricular white matter regions, concomitant parenchymal infiltration, and lymphocytes’ accumulation around small vessels [180]. The occurrence of possible vasculitis-induced ischemic changes has also been claimed in a patient with chronic HCV infection, who developed skin vasculitis and leukoencephalopathy over 3 years [181]. The spectrum of cognitive alterations in HCV patients is not limited to the preceding vasculitic forms but includes inflammatory disorders, such as acute encephalitis, encephalomyelitis, and meningoradiculitis/polyradiculitis [182]. There are reports of patients with rapidly evolving leukoencephalitis with microglial nodules. Perivascular T-cell infiltrates in association with HCV genome presence [39] or fatal progressive encephalomyelitic syndromes, pathologically characterized by neuronal loss and perivascular lymphocyte cuffing in the brainstem and cervical spinal cord [42]. In these cases, available evidence suggests the occurrence of an immune-mediated process induced by HCV rather than a direct effect of the virus [182]. However, there is growing evidence that there are fundamental cognitive deficits in many patients with HCV before developing cirrhosis unrelated to liver dysfunction indices, viral load, or genotype [182]. Recently many aspects indicated subtle frontal cognitive impairment in subclinical HE [183]. Additionally prominent among patients with HCV are complaints of problems with thinking that have been described as “brain fog” or problems with attending to and recalling everyday information and executive functions [183]. Proton magnetic resonance spectroscopy [184] found a correlation between these neuropsychological findings and cerebral metabolic abnormalities in the frontal white matter and basal ganglia of HCV patients with little or no fibrosis [185]. A well-conducted study on pre-pulse inhibition, a measurement of attentional processing [186], demonstrated that in HCV patients without HE, the performances were definitively lower than normal controls. The neural networks that sustained this cerebral attention process highly rely upon the forebrain-cortico-striatal-pallidal-thalamic circuit [187].

HCV patients are fragile subjects, with many interfering factors, including premorbid psychiatric conditions, lifestyle events, comorbid psychiatric diseases (i.e., depression, substance addictions, iatrogenic disorders, i.e., interferon employment, etc.). Nevertheless, with all of these aspects considered, whenever we are making a hypothesis on HCV-related cognitive disturbances, chronic activation of the immune system may hold importance. Once individuals are infected with HCV, cytokines such as interleukin (IL)-2, IL-4, IL-10, and interferon-gamma are produced [188]. They may continue to be elevated for as many as 20–30 years and longer, inducing blood–brain alterations or constant microglia activation [189]. Cytokines are postulated to indirectly affect brain functioning by transmitting signals via the vagus nerve or other visceral afferent neuronal pathways and binding to the cerebral vascular endothelium, and inducing secondary messengers prostaglandins and nitric oxide [189,190,191]. Microglia are known to release excitatory amino acids that can cause neuronal cell death via excitotoxicity, and they can exert a neuro-modulatory role, i.e., glutamate neurotoxicity [192]. The basal ganglia neural networks are more prone to suffer with altered dopamine-transporter binding, particularly impaired meso-telencephalic dopamine projections to the frontal lobe [193].

## 8. Conclusions

There is clear evidence that HCV infection is related to the development of neurological and neurodegenerative disorders, which mainly depend on two factors: (1) extra-hepatic replication of HCV with related local activation of the inflammatory and (2) HCV effect on the immune system and autoantibody synthesis. Nevertheless, clinical and virologic studies are still needed to fully understand the intricate relationship between HCV and nervous system tissues and how clinical manifestations may develop.

## Data Availability

Not applicable.
